# Flexible pri-miRNA structures enable tunable production of 5’ isomiRs

**DOI:** 10.1080/15476286.2022.2025680

**Published:** 2022-02-19

**Authors:** Xavier Bofill-De Ros, Zhenyi Hong, Ben Birkenfeld, Sarangelica Alamo-Ortiz, Acong Yang, Lisheng Dai, Shuo Gu

**Affiliations:** a RNA Mediated Gene Regulation Section, RNA Biology Laboratory, Center for Cancer Research, National Cancer Institute, Frederick, MD, USA; b Neural Development Section, Mouse Cancer Genetics Program, Center for Cancer Research, National Cancer Institute, Frederick, MD, USA

**Keywords:** Drosha, dicer, cleavage fidelity, alternative cleavage, 5’ isomiRs, pri-miRNA, RNA structure, RNA-binding proteins

## Abstract

The Drosha cleavage of a pri-miRNA defines mature microRNA sequence. Drosha cleavage at alternative positions generates 5’ isoforms (isomiRs) which have distinctive functions. To understand how pri-miRNA structures influence Drosha cleavage, we performed a systematic analysis of the maturation of endogenous pri-miRNAs and their variants both *in vitro* and *in vivo*. We show that in addition to previously known features, the overall structural flexibility of pri-miRNA impact Drosha cleavage fidelity. Internal loops and nearby G · U wobble pairs on the pri-miRNA stem induce the use of non-canonical cleavage sites by Drosha, resulting in 5’ isomiR production. By analysing patient data deposited in the Cancer Genome Atlas, we provide evidence that alternative Drosha cleavage of pri-miRNAs is a tunable process that responds to the level of pri-miRNA-associated RNA-binding proteins. Together, our findings reveal that Drosha cleavage fidelity can be modulated by altering pri-miRNA structure, a potential mechanism underlying 5’ isomiR biogenesis in tumours.

**Graphical Abstract:**
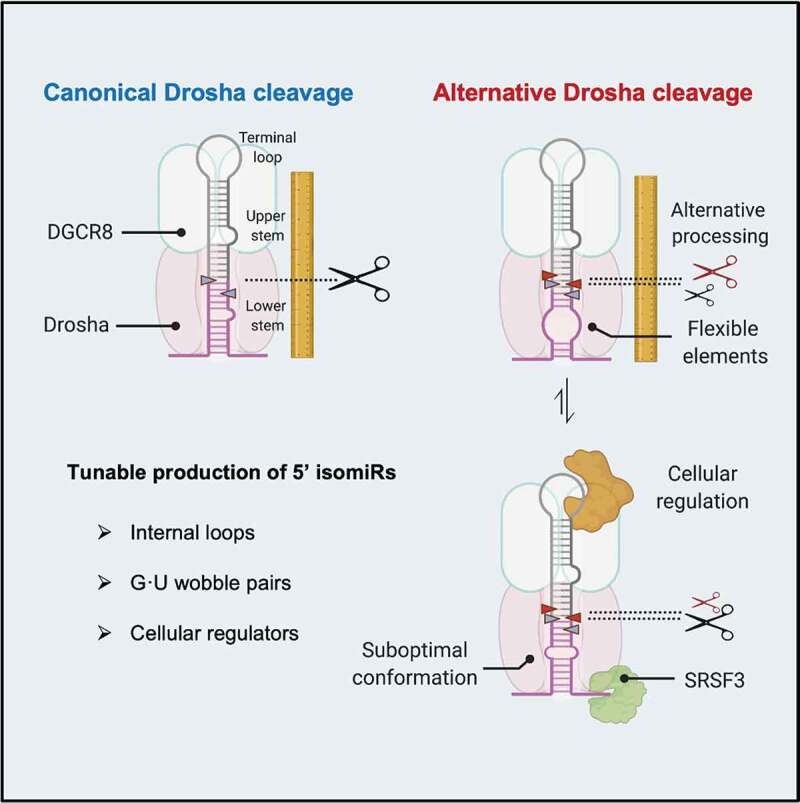

## Introduction

**Figure 1.**
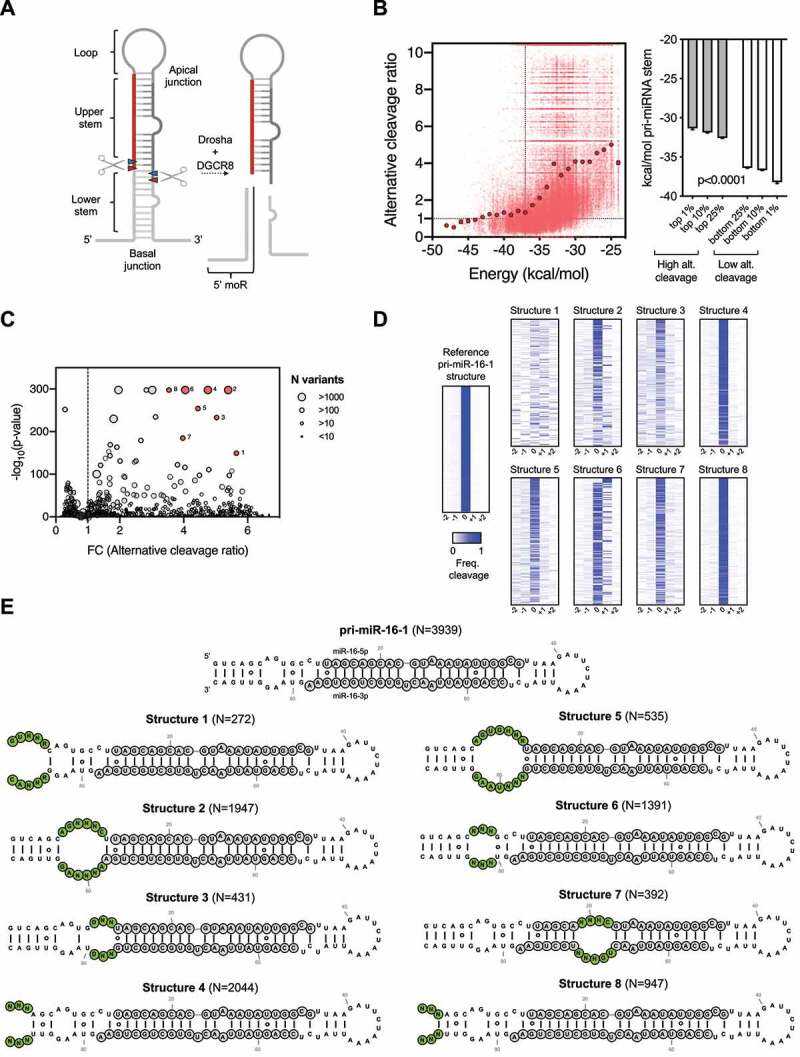
More flexible pri-miRNA structure correlates with alternative Drosha cleavages.

MicroRNAs (miRNAs) play a critical role in cellular physiology by inhibiting gene expression via post-transcriptional mechanisms [1]. Misregulation of miRNAs is involved in the pathogenesis of many diseases including cancer [2]. MiRNAs are transcribed as part of longer transcripts, primary miRNA (pri-miRNAs). Pri-miRNAs fold into hairpin structures, which are cleaved by Drosha into precursor miRNA (pre-miRNA). Dicer cleaves pre-miRNAs in the cytoplasm [3]. The resulting RNA duplexes (∼22 nt) are loaded onto Argonaute proteins. Eventually, one of the two strands remains associated with the Argonaute protein, forming the core of the RNA-induced silencing complex (RISC) [4,5]. In metazoans, miRNA guides RISC to target mRNAs by partial base-pairing, providing inhibition specificity [6,7]. The sequence of nucleotides from position 2 to 8, counting from the miRNA 5’ end, plays a crucial role in this process. Base-pairing of this small ’seed’ region with targets is required and often sufficient for a miRNA to function [8]. Ends of miRNAs are defined by Drosha and Dicer cleavages. Drosha cutting at an alternative position generates miRNA isoforms (isomiRs) with distinct 5’ and 3’ ends, on 5p and 3p miRNAs respectively (Fig. 1A). As a consequence, 5’ isomiRs have altered seed sequence, while 3’ isomiRs have potentially changed supplemental pairing, leading to a reshaped target repertoire [1]. This highlights the importance of understanding the mechanisms by which Drosha cleavage fidelity is governed.

(A) Scheme of the pri-miRNA structure. Red and blue arrows indicate Drosha cleavage sites, which can be measured from a sequence of the 5’ moR products. (B) Left, scatter plot of the alternative cleavage ratio (relative to pri-miR-16-1 wild-type sequence) as a function of the minimum folding energy (kcal/mol) of each sequence variant. Right, the minimum folding energy (kcal/mol) of pri-miR-16-1 sequence variants with high and low alternative cleavage levels. (C) Average alternative cleavage ratios of pri-miR-16-1 sequence variants with the same predicted secondary structure were plotted against the p-values calculated by comparison with the variants with the same secondary structure as the pri-miR-16-1 wild-type (N = 3939) using the Wilcoxon test. The size of the dots indicates the number of sequence variants supporting that structure group. (D) Heatmaps displaying the frequency of cleavage at different positions along the pri-miRNA stem for each structure group, being 0 the canonical cleavage site (200 sequence variants are shown for each structure). (E) Structural variants with the most significant increases in alternative Drosha cleavage compared to variants with the native structure. N indicates the number of sequence variants supporting that structure group. Nucleotides involved in the structural feature are indicated in green and shown in the IUPAC nucleotide code (N = [A/G/C/U], H = [A/C/U], D = [A/G/U], R = [A/G]).

Drosha and its cofactor DGCR8 form a complex called microprocessor, which determines its cleavage site by recognizing a set of structural and sequence features of pri-miRNA. The pri-miRNA hairpin is structurally defined by a terminal loop (8–38nt), a stem with a high degree of complementarity (~35 bp) and unstructured flanking sequences [9]. The stem is divided into an upper stem where the mature miRNA sequence resides and a lower stem next to the flanking regions [10]. Drosha senses the basal junction between the flanking regions and the lower stem, and cleaves ~11 bp away [11]. A DGCR8 dimer binds to the apical junction between the upper stem and terminal loop, facilitating the recognition of pri-miRNAs [10,12]. Although the Drosha-DGCR8 complex is sufficient to process most pri-miRNAs *in vitro* [13], recent findings indicate that Drosha cleavage is modulated by a large number of pri-miRNA associated RNA-binding proteins (RBPs) *in vivo* [14]. Several sequence motifs, including a ‘UG’ at the basal junction, a ‘UGU’ at apical junction and ‘CNNC’ at the flanking region, are recognized by Drosha, DGCR8 and SRSF3, respectively [15–17].

While all these structural and sequence features contribute to efficient Drosha processing, the extent to which these features impact Drosha cleavage fidelity remains unclear. Several studies have shown that stem length affects Drosha cleavage fidelity by defining the relative distances between the expected cleavage site, the basal junction, and the apical junction of a pri-miRNA [18–20]. Furthermore, an interaction between Drosha dsRBD and a mGHG motif in the lower stem promotes precise cleavage by facilitating the alignment between Drosha and pri-miRNA substrates [15,17]. More recently, mismatched and wobbled base pairs along the upper stem were found to impact the Drosha cleavage fidelity as well [21]. However, these mechanisms, individually or in combination, cannot fully account for the extent of alternative Drosha cleavage observed in cells. Furthermore, it is difficult to explain why Drosha cleavage fidelity on a given pri-miRNA can differ in different cells [22,23] if it is determined only by these invariable features. Therefore, additional RNA elements that are amenable to regulation might contribute to Drosha cleavage fidelity.

By studying Drosha processing of the human pri-miR-9 family, we showed previously that the distorted and flexible structures of the pri-miR-9-1 lower stem promotes the Drosha cleavage at an alternative site [22]. Pri-miR-9-2 and pri-miR-9-3, despite encoding the same mature miRNA, are cleaved by Drosha at a single site. As a result, a 5’ isomiR (miR-9-5p-alt, iso_5p:+1) is exclusively generated from pri-miR-9-1 and regulates a distinctive set of target genes in low-grade glioma. The case study of pri-miR-9 provided the first evidence linking pri-miRNA tertiary structure with the Drosha cleavage fidelity. However, it remains unclear to what extent this applies to pri-miRNA processing in general.

Here, by analysing *in vitro* Drosha cleavages of over 210,000 variants of pri-miR-16-1, pri-miR-30a and pri-miR-125a, we systematically investigated the relationship between pri-miRNA structure and Drosha cleavage fidelity. We report that the structural flexibility introduced by unpaired regions and nearby G:U wobble pairs along the pri-miRNA stem leads to increased alternative cleavage of Drosha, which in turn impact the Dicer processing and hence contributes to 5’ isomiR production from both 5’ and 3’ arms of a pre-miRNA. Furthermore, we performed hypothesis-driven mutagenesis on pri-miR-9 and validated these conclusions on Drosha processing in cells. By analysing data deposited in the Cancer Genome Atlas (TCGA), we provide evidence that alternative cleavage of pri-miRNAs is a tunable process that responds to the levels of pri-miRNA-associated RBPs. Together, our findings reveal that Drosha cleavage fidelity can be modulated by altering pri-miRNA structure, a mechanism by which cells might regulate 5’ isomiR biogenesis in tumours.

## Results

### More flexible pri-miRNA structure correlates with alternative Drosha cleavages

To study how pri-miRNA structure impacts Drosha cleavage fidelity, we took advantage of a published dataset [15] originally used to identify motifs required for efficient Drosha processing. Pri-miR-16-1, pri-miR-30a, pri-miR-125a and >210,000 variants with various sequences mutated along the pri-miRNA stem were cleaved by Drosha *in vitro*. By analysing the sequences of the cleavage products, specifically the 5’ miRNA-offset RNA (5’ moR) [24], we observed on average ~150 cleavage events per variant and determined the corresponding Drosha cleavage site (Fig. 1A). Consistent with previous reports [17,18,22,24,25], a portion of Drosha cleavages occurred at positions other than the canonical site for all three pri-miRNAs (Sup. Fig. 1A), indicating that alternative Drosha processing is an intrinsic phenomenon.

We predict the minimum-free energy (MFE) of each pri-miR-16-1 variant and use it as an indication of the overall structural flexibility. Variants less structured (more flexible) than the wild-type pri-miR-16-1 (−37 kcal/mol) were processed by Drosha with an alternative cleavage frequency 2 to 6 times higher (Fig. 1B) indicating that the structural flexibility of pri-miRNA is inversely correlated with Drosha cleavage fidelity. Supporting this idea, pri-miR-16-1 variants with the highest alternative cleavage rates (top 1%) had the lowest average MFE value, while those with the lowest alternative cleavage rates (bottom 1%) had the highest average MFE value (Fig. 1B). The same analysis with pri-miR-30a, pri-miR-125a and their variants generated similar results (Sup. Figs. 1B–E), demonstrating that more flexible pri-miRNA structures were processed by Drosha with a lower cleavage fidelity.

Next, we sought to identify structural features that impact Drosha's alternative cleavage. To this end, we grouped pri-miR-16-1 sequence variants (~80,000) based on their predicted secondary structures (N = 1308 structural variants) with each group sharing the same structure. The average alternative Drosha cleavage ratio of each structural group was calculated and then compared to that of a group resembling wild-type pri-miR-16-1 structure (Fig. 1C). While many structural groups showed increased alternative cleavage, we focused on those with extremely low p-values (<10^−150^), because variants within these groups had a rather consistent perturbation of their cleavage fidelity regardless of their sequence variations. Further analyses confirm that this is indeed the case for these structural groups (Fig. 1D). It is suggested that these particular structures but not their sequences per se, underlie the increased alternative cleavage (Fig. 1D). We selected the top eight structural variants with the highest fold change of alternative cleavage and lowest p-value for further characterization (Fig. 1E). Three of them (structures 1, 4 and 8) presented a disruption of the basal junction, while variant 6 contained an unpaired region at the position of the mGHG motif, confirming the critical role of both basal junction [18] and mGHG motif [15,17] in determining the Drosha cleavage site. We also validate our approach by identifying pre-miRNA structures that promote alternative Drosha cleavage. On the other hand, structural variants 2, 3, 5 and 7 cannot be explained by the current model but nonetheless lead to alternative Drosha cleavage. After measuring their tertiary structure differences by the root-mean-square deviation (RMSD), we found that they all had relatively high variation compared to pri-miR-16-1 (WT) (Sup. Fig. 1F). The same analysis on pri-miR-30a and pri-miR-125a datasets generated similar conclusions (Sup. Figs. 1G and 1H). Consistent with the insights gained from the case study of pri-miR-9 [22], these results indicate that besides previously identified structural features, the overall distortion, and flexibility of the pri-miRNA stem also determines to a large extent the alternative Drosha cleavage.

### Unpaired internal loops lead to alternative Drosha cleavages

**Figure 2.**
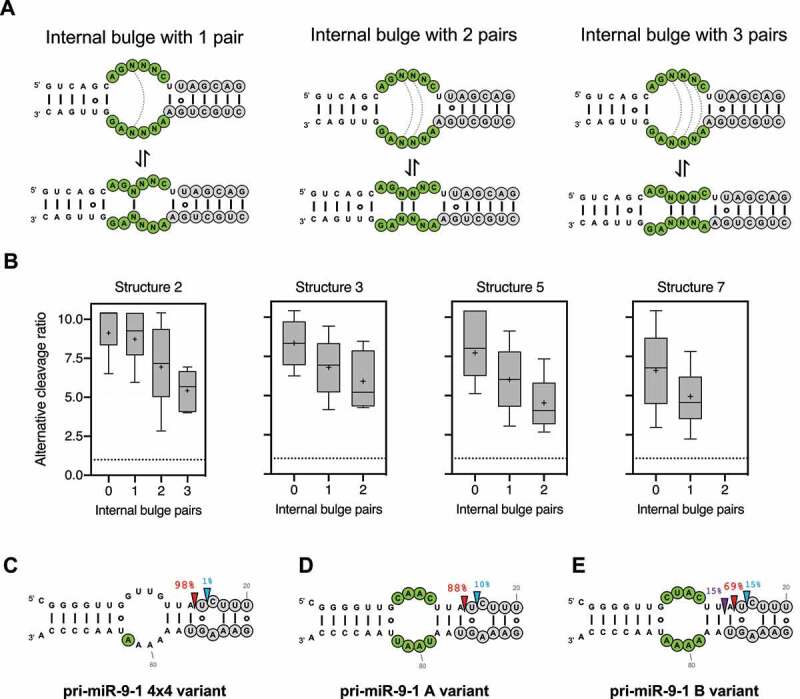
Unpaired internal loops lead to alternative Drosha cleavages.

The four pri-miR-16-1 structural variants identified with a high ratio of alternative cleavage all contained a relatively large internal bulge along the stem (Fig. 1D). It is possible that these internal bulges are partially closed via RNA ‘breathing’ motions, reducing overall structural flexibility, which in turn impacts alternative Drosha cleavage. To test this we further classified sequence variants within each structure based on the number of potential base-pairs formed in the internal loop during these transient rearrangements (Fig. 2A). For all structural variants tested, we observed reduced alternative Drosha cleavage when the internal loop could be partially paired (Fig. 2B). The same results were obtained with a similar analysis of pri-miR-30a and pri-miR-125a (Sup. Fig. 2A), suggesting that pri-miRNA tertiary structural flexibility associated with unpaired regions causes alternative Drosha cleavage *in vitro*.

(A) Scheme of the internal bulge of pri-miR-16-1 structural variant 2 are in equilibrium with structures that can close the bulge with one, two or three potential base-pairs. (B) Box plots with the alternative cleavage ratio of sequence variants that can form different numbers of base-pairs in the structural variant groups 2, 3, 5 and 7, previously described in Fig. 1D. (C, D, E) HEK293T cells transfected with plasmids expressing pri-miR-9-1 lower stem variants. Nucleotides that change from the wild-type sequence are indicated to be green. Small RNAs were subjected to deep sequencing. After being mapped to the corresponding pri-miR-9 structure, the percentage of sequences starting at a position relative to the total number of miR-9 reads was used to infer the Drosha site cleavage percentage. Canonical and alternative cleavage sites are indicated with a red, blue and purple arrow, respectively.

To test this in living cells, we took advantage of pri-miR-9-1, the Drosha processing of which has been well characterized: an asymmetrical internal bulge (4x3) at the lower stem of pri-miR-9-1 is responsible for its alternative Drosha cleavage (~15%) which can be reduced to <1% by correcting the asymmetry [22]. The resulting symmetrical bulge (4x4) can form two internal base-pairs (Sup. Fig. 2B), suggesting this partial pair as the source of reduced alternative Drosha cleavage (Fig. 2C). To test this we disrupted the internal pairs by mutagenesis and measured their Drosha cleavage in HEK293T cells by deep sequencing. The resulting pri-miR-9-1 mutants while maintaining the 4 × 4 symmetrical bulge, had greater flexibility (Sup. Fig. 2C). As expected, we observed substantial amounts of alternative Drosha cleavage (Fig. 2D and E). Together, these results demonstrate that unpaired bulges contribute to pri-miRNA structural flexibility, which in turn leads to alternative Drosha cleavage both *in vitro* and *in vivo*.

### G · U wobble pairs contribute to alternative Drosha cleavage by enhancing pri-miRNA structural flexibility

**Figure 3.**
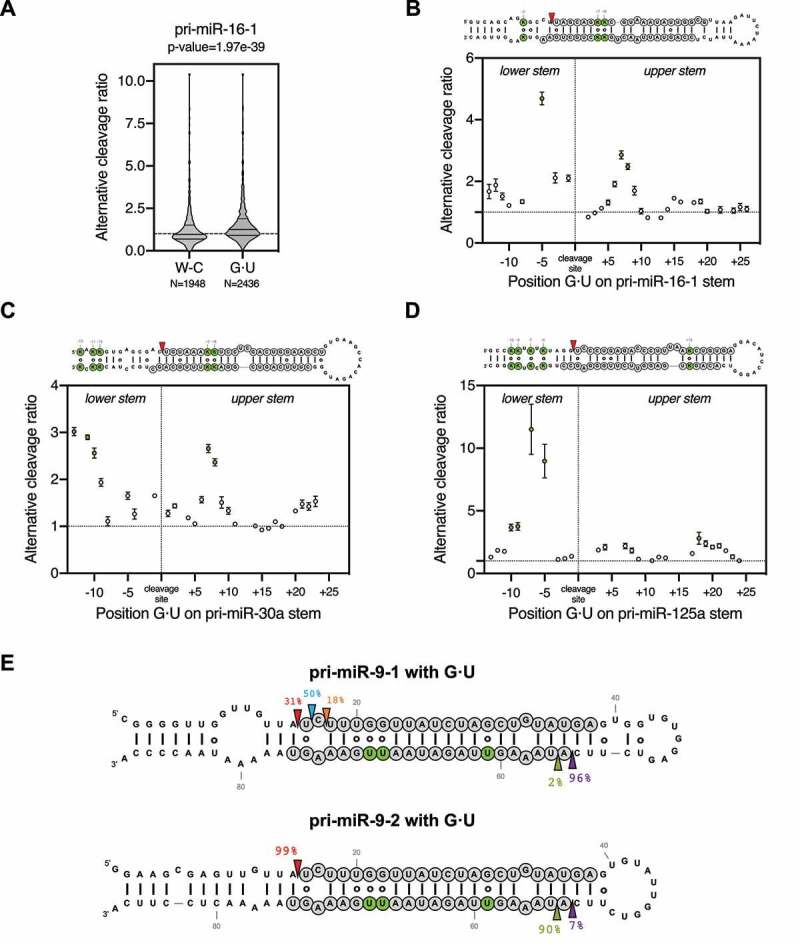
G·U wobble pairs contribute to alternative Drosha cleavage by enhancing pri-miRNA structural flexibility.

G · U pairs are known to disrupt the dsRNA A-form helix structure [26]. Therefore, we sought to investigate how they contribute to the structure-flexibility-mediated alternative Drosha cleavage. To this end, we analysed all pri-miR-16-1 sequence variants that are predicted to have the same secondary structure but different numbers of G · U pairs compared to the wild-type pri-miR-16-1. Compared to variants that have the same number of G · U pairs as the wild-type (N = 1,948 sequence variants), those with additional G · U pairs (N = 2,436 sequence variants) were processed by Drosha with a higher average alternative cleavage ratio (Fig. 3A). The same analysis for pri-miR-30a and pri-miR-125a variants showed a similar result (Sup. Fig. 3), indicating that G · U pairs promote alternative Drosha cleavage. However, the average effect is subtle with a large variation, suggesting that not all G · U pairs contribute equally. We analysed the impact of the G · U position on alternative cleavage and found that changes at certain positions had a much larger effect (Fig. 3B). The positions of these hotspots were not consistent among pri-miR-16-1, pri-miR-30a and pri-miR-125a, suggesting that the effect is unlikely to be caused by specific interactions between Drosha and the pri-miRNA substrate. Instead, incorporating G · U pairs at positions near existing bulges increased the alternative cleavage rate (Fig. 3B–D), suggesting that the G:U pairs promote alternative Drosha cleavage by enhancing existing structural flexibility.

(A) Violin plot of the alternative cleavage ratio of pri-miR-16-1 variants with wild-type native structure. W-C indicate variants with the same number of G · U pairs as the wild-type, and G · U indicates variants with additional G · U pairs. (B, C, D) Analysis of the effect of the addition of single G · U wobble pairs on alternative cleavage based on their position in the stem of pri-miR-16-1, pri-miR-30a and pri-miR-125a (dots indicate mean and standard error). Secondary structures indicate in green the location of G · U wobble pairs with the largest impact on alternative Drosha cleavage. (E) HEK293T cells transfected with plasmids expressing either pri-miR-9-1 or pri-miR-9-2 variants with additional G · U wobble pairs (indicated in green). Small RNAs mapping to each pri-miRNA were used to infer the percentage of Drosha and Dicer cleavage at each position. The canonical cleavage sites of Drosha and Dicer are indicated with red and purple arrows, while blue, orange and green indicate alternative cleavage sites.

To test this idea, we took advantage of pri-miR-9-1 and pri-miR-9-2, with the former having a distorted tertiary structure, and the latter being structurally rigid [22]. Replacing multiple Watson-Crick G-C pairs by G · U pairs along the stem significantly increased the alternative cleavages of pri-miR-9-1 but had no effect on pri-miR-9-2 when expressed in HEK293T cells (Fig. 3E). Together, these results demonstrate that a G:U pair per se has minimal impact on Drosha's cleavage. However, G:U wobble pairs contribute to alternative Drosha cleavage by enhancing the structural flexibility of pri-miRNAs.

### Alternative Drosha cleavage results in 5’ isomiRs from both strands of pre-miRNAs

**Figure 4.**
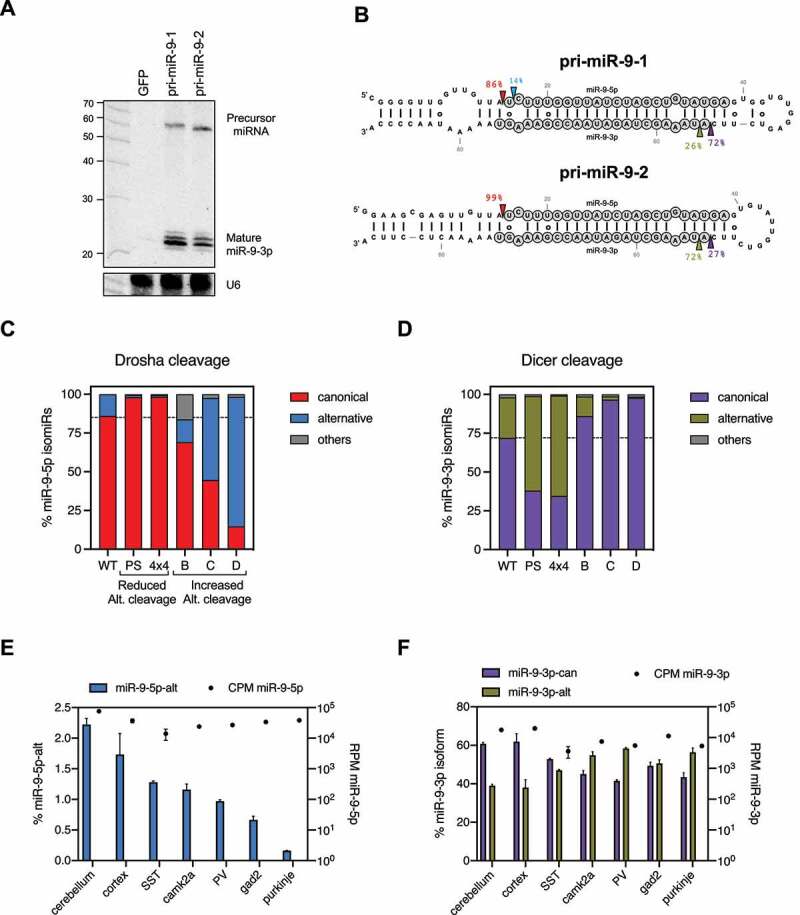
Alternative Drosha cleavage results in 5’ isomiRs from both strands of pre-miRNAs.

Drosha and Dicer alternative cleavages generate 5’ isomiRs from the 5p arm and 3p arm of pre-miRNAs respectively (Sup. Fig. 4A). Given that Drosha processing determines to a large extent where Dicer cuts [27,28], alternative Drosha cleavage may also contribute to the production of 3p isomiRs. To test this we expressed pri-miR-9-1 and pri-miR-9-2 separately in HEK293T cells and examined the biogenesis of their 3p isomiRs. Northern blot analysis revealed that both pri-miR-9 transcripts produced 3p isomiRs (miR-9-3p) (Fig. 4A). Using deep sequencing, we found that these miR-9-3p reads were composed of two populations: a canonical miR-9-3p (miR-9-3p-can) as annotated by the miRBase/MirGeneDB and a 5’ isomiR (miR-9-3p-alt, iso_5p:+1) that begins one nucleotide downstream (Fig. 4B). Interestingly, their relative abundances differed between pri-miR-9-1 and pri-miR-9-2. While miR-9-3p-can was the dominant isomiR processed from pri-miR-9-1 (~70%), it only accounted for ~30% of reads generated from the pri-miR-9-2 (Fig. 4B). These results indicate that the dominant Dicer cleavage site on pri-miR-9-1 is one nucleotide upstream of the Dicer cleavage site on pri-miR-9-2. Because the current model indicates that the Dicer cuts at a fixed distance from where Drosha cuts [28–30], this shift of the Dicer cleavage position aligns well with the alteration of Drosha cleavage sites (Fig. 4B). This suggests that the decreased amount of miR-9-3p-alt generated from pri-miR-9-1 is likely a result of its alternative Drosha cleavage. To test this we examined the biogenesis of 3p isomiRs in multiple pri-miR-9-1 mutants, all of which have the same upper stem and loop sequence but different Drosha cleavage patterns due to variation in the lower stem sequence (Fig. 4C and Sup. Fig. 4B). As expected, reduced alternative Drosha cleavage led to higher levels of miR-9-3p-alt, whereas increased alternative Drosha cleavage resulted in lower levels of miR-9-3p-alt (Fig. 4D). Together, these results demonstrate that Drosha processing contributes to the production of 3p isomiRs by impacting the Dicer cleavage.

(A) HEK293T cells transfected with plasmids expressing either pri-miR-9-1 or pri-miR-9-2 were subjected to northern blotting to detect miR-9-3p expression. (B) Small RNAs mapping to each pri-miRNA paralog were used to infer the percentage of Drosha and Dicer cleavage at each site. (C, D) Frequency of reads with the canonical or alternative 5’ end for miR-9-5p (Drosha cleavage) and miR-9-3p (Dicer cleavage) for different pri-miR-9-1 variants on the lower stem. Canonical and alternative 5’ ends were defined based on miRBase/MirGeneDB annotation for the mature miRNA [9,46,47]. (E, F) Small RNA deep sequencing data from different mouse brain tissues (cerebellum, cortex) and cell-types (Purkinje cells, Camk2α cells, parvalbumin (PV) neurons and neuropeptide somatostatin (SST) neurons) were re-analysed to assess miR-9-5p and miR-9-3p isoforms (left axis) and overall expression (RPM, counts per million, right axis).

To extend our conclusions beyond cultured cells, we examined miR-9 biogenesis in mouse tissues. Consistent with previous studies, miR-9 expression was highly specific to the brain (Sup. Fig. 4C). Analyses of a previously published sRNA-seq dataset [31] revealed that miR-9 is expressed in multiple brain tissues and various neuronal cells. Despite the rather homogenous expression level (~40,000 CPM), the percentage of the 5p isomiR (miR-9-5p-alt) ranged from 2.2% in cerebellum to less than 0.2% in Purkinje cells (Fig. 4E), suggesting variations in alternative Drosha cleavages. The distribution of 3p isomiRs was changed accordingly: a higher percentage of miR-9-3p-can was observed in tissues/cells with a higher percentage of miR-9-5p-alt, while more miR-9-3p-alt was observed in those with a lower percentage of miR-9-5p-alt (Fig. 4F), which is consistent with the pattern observed in HEK293T cells. These results suggest that the alternative Drosha cleavage could play a biological role in regulating 5’ isomiR biogenesis.

### Alternative Drosha cleavage is subjected to cellular regulation

**Figure 5.**
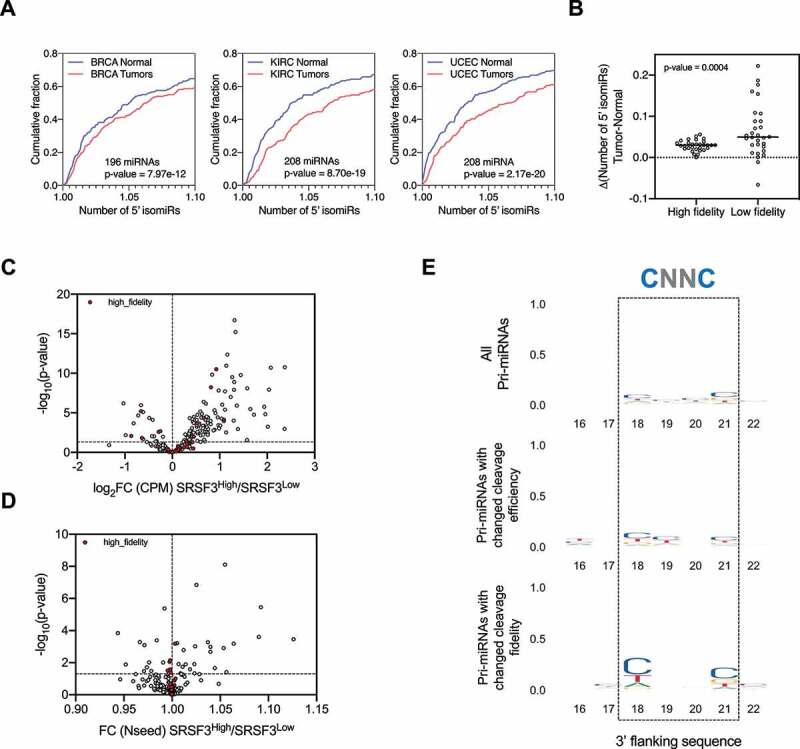
Alternative Drosha cleavage is subjected to cellular regulation.

Drosha cleavage fidelity on a given pri-miRNA varies among cell types [22,23], suggesting cellular regulation. To further test this idea, we took advantage of the Cancer Genome Atlas (TCGA), where a large amount of miRNA-seq and corresponding RNA-seq data are available. We measured the relative levels of 5’ isomiRs for the top 200 abundant miRNAs in 1015 Breast Invasive Carcinoma (BRCA) samples. Comparing these to normal tissue controls (N = 104), we observed a subtle but significant increase in 5’ isomiRs. We made a similar observation was made with samples obtained from Kidney Renal Clear Cell Carcinoma (KIRC) and Uterine Corpus Endometrial Carcinoma (UCEC) patients, indicating that the upregulation of 5’ isomiRs is not limited to one type of cancer (Fig. 5A). These results suggest that Drosha's processing is altered during tumorigenesis, resulting in 5’ isomiRs that could potentially impact tumour progression. Interestingly, pri-miRNAs cleaved by Drosha with high fidelity in normal cells were more resistant to such an alteration whereas pri-miRNAs with low Drosha cleavage fidelity were more prone to the increase in alternative Drosha cleavage (Fig. 5B).

(A) The relative number of 5’ isomiRs was measured using an inverted Simpson diversity index. Using this measurement, we analysed all highly expressed miRNAs by comparing normal and primary tumour samples from BRCA, KIRC and UCEC. The results are plotted as a cumulative distribution for all miRNAs between normal and tumoural samples. (B) Delta of the number of 5’ isomiRs between BRCA primary tumours and normal samples. The high fidelity and low fidelity groups were defined as the top and bottom 30 miRNAs, out of the top 100 most expressed, according to their levels of 5’ isomiRs in normal samples. (C) Volcano plot of the changes in mature miRNA expression between the two sets of BRCA primary tumour samples with high and low levels of SRSF3 expression (N = 100 samples/group). A group of miRNAs processed with high fidelity (defined in Fig. 5B) is shown in red. (D) Similarly, volcano plot of the changes in mature miRNA Number of 5’ isomiRs (Nseed) between BRCA primary tumours with high/low levels of SRSF3. (E) Motif logo of the 3’ flanking sequence of the following groups: all pri-miRNAs included in the analysis (upper panel), pri-miRNAs with differential cleavage efficiency (miRNA expression) (FC>1 and p < 0.05) (middle panel), pri-miRNAs with differential cleavage fidelity (p < 0.05) (lower panel). Box indicates the position for binding of SRSF3 previously described.

Given the role that structure plays in defining Drosha cleavage sites, the regulation of 5’ isomiR production could be achieved, at least in part, via modulating pri-miRNA structures by association of RBPs. To test this we sought to investigate whether SRSF3, an RBP known to bind to pri-miRNAs at a ‘CNNC’ motif downstream of the Drosha cleavage site, could impact alternative Drosha cleavages. To this end, we compared the miRNA profiles between BRCA patients with high levels of SRSF3 (top 10%, 100 samples) and relatively low levels of SRSF3 (bottom 10%, 100 samples). Consistent with the known role of SRSF3 in promoting miRNA biogenesis [16,32], expression levels of most miRNAs were higher in samples with a higher level of SRSF3, confirming our approach (Fig. 5C). The fidelity of Drosha cleavage on a subset of pri-miRNAs also varied between the two groups (Fig. 5D and Supplementary Table 1). A ‘CNNC’ motif was enriched in this subset of pri-miRNAs, indicating that the observed changes in Drosha fidelity were likely a direct effect of SRSF3 association (Fig. 5E and Sup. Fig. 5A).

Similar analyses of a set of RBPs known to associate with pri-miRNAs [14,33] generated similar results, whereas the level of randomly selected RBP RBM22 as well as a non-RBP Tubulin (TUBA1A), had marginal, if any effects on 5’ isomiR profiles (Sup. Fig. 5B). Different RBPs had different impacts: while a higher level of DDX3X, DDX21, hnRNPA1 or hnRNPH2 generally promoted alternative Drosha cleavage (Sup. Fig. 5C), FUS and hnRNPH1 apparently prevented Drosha alternative processing (Sup. Fig. 5D). In all cases, pri-miRNAs with no change in Drosha cleavage fidelity during tumorigenesis also showed no impact from these RBPs, indicating that RBP-mediated regulation of alternative Drosha cleavage is limited to those pri-miRNAs without a well-defined Drosha cleavage site.

## Discussion and conclusion

As an initial step in licencing miRNA production, Drosha cleavage of pri-miRNAs has been extensively studied. A comprehensive set of structural and sequence features of pri-miRNAs have been identified to play an important role in determining Drosha cleavage efficiency and fidelity. However, most endogenous pri-miRNAs have only a subset of these features, suggesting certain evolutionary advantages to having non-optimal processing. Here, by analysing tens of thousands of *in vitro* Drosha cleavage events, we provide robust statistical evidence indicating that pri-miRNA structural flexibility introduced by internal loops and G:U wobbles is positively correlated with alternative Drosha cleavage. Using the mutagenesis study of pri-miR-9, we established causality and validated these conclusions in living cells. Pri-miRNA structural flexibility, different from previously identified features, is amenable to cellular regulation. Indeed, we provide evidence that the level of a set of pri-miRNA binding proteins, including SRSF3, correlates with the use of alternative Drosha cleavage sites in tumours. Given the prevalence of internal bulges and G:U wobbles along the pri-miRNA stem, our findings support a model in which Drosha cleavage fidelity is regulated by modulating pri-miRNA structure via association with RBPs.

It is intriguing to speculate why pri-miRNAs with distorted stems are processed by Drosha with a lower cleavage fidelity. It is possible that the higher flexibility of pri-miRNAs enables them to fold into several distinct suboptimal structures when complexing with Drosha. In this case, the various cleavage sites may be a result of different configurations of the catalytic centre and substrate. Indeed, the flexible lower stem of pri-miR-9-1, which has a ~ 15% chance to be cut by Drosha at an alternative site, can fold into two suboptimal structures (Sup. Fig. 6A). Two constructs (SUB1 and SUB2) designed to mimic these suboptimal conformations were processed by Drosha differently: Drosha cut SUB1 primarily at the canonical site (Sup. Fig. 6B), whereas SUB2 was processed with a higher level of miscleavage than the wild-type structure (Sup. Fig. 6C). This suggests that the overall Drosha cleavage profile of pri-miR-9-1 may be an ensemble of different configurations between Drosha and two suboptimal folds of pri-miR-9-1. Future high-resolution structures of the ternary complex formed by Drosha, DGCR8 and pri-miRNA should give additional insights [34,35].

Using chemical probing approaches, a recent report measured the endogenous RNA structures on a genome-wide scale [36]. When comparing RNAs extracted from different cellular compartments, the authors found that RNA structures vary less *in vitro* than *in vivo*. In particular, conserved pri-miRNAs form relatively stable structures *in vitro* yet are highly influenced by cellular factors, resulting in different RNA-folds *in vivo*. A follow-up study demonstrated that these distinct structures of pre-miRNAs correlate with the Dicer cleavage efficiency and fidelity [37]. Furthermore, high-throughput studies analysing pri-miRNA processing *in vitro* and *in vivo* found that cleavage fidelity is regulated *in vivo* by RBPs, such as SRSF3 [38–40]. These results further support our model that pri-miRNA structure is modulated to fine-tune 5’ isomiR production. We have previously shown that a Drosha isoform lacking the nuclear localization signal due to alternative splicing can process a subset of pri-miRNAs in the cytoplasm [41]. It is possible that the cytoplasmic pri-miRNAs are processed with different Drosha cleavages fidelity due to changes in their structures. This might help to explain why up-regulation of cytoplasmic Drosha coincides with misregulation of miRNAs in tumours.

5’ isomiRs resulting from alternative Drosha cleavages play diverse biological roles. In particular, we have shown that 5’ isomiRs of miR-9-5p processed from pri-miR-9-1 regulates a distinct set of target mRNAs in low-grade gliomas [22]. Another study has shown that miR-9-3p has a critical role in hippocampal synaptic plasticity and memory [42]. It is possible that the 5’ isomiR of miR-9-3p also plays a functional role in brain cells where it is differentially expressed. Here, we find that Drosha cleavage fidelity decreases in multiple cancers, resulting in numerous miRNAs with altered 5’ ends and seed sequences. This led us to hypothesize that Drosha processing is altered during tumorigenesis to produce 5’ isomiRs that impact tumour progression. Our findings show that pri-miRNA-associated RBPs promote or inhibit 5’ isomiR production by modulating the pri-miRNA structure, providing one possible underlying mechanism. It is possible that only a subset of the aberrant 5’ isomiRs found in tumours have a functional role. Future studies identifying oncogenic or tumour suppressive 5’ isomiRs will provide additional insights.

## Supplementary Material

Supplemental Material

## Data Availability

Small RNA-seq data is deposited at GEO with the accession number GSE172446 [Reviewer access: ijobkaeshbmtfcd] and GSE108893. As well as previously published Ago2-IP in mouse brain (GSE30286). The Cancer Genome Atlas (TCGA) can be accessed via dbGaP study accession: phs000178.v11.p8.

## Cell lines

HEK293T cells were maintained in DMEM high glucose (Gibco) supplemented with 10% heat-inactivated foetal bovine serum (Hyclone), 100 U/ml penicillin-streptomycin (Gibco) at 37°C. The cells were tested to be free of mycoplasma contamination. Transfections were performed using PolyJet™ DNA Transfection Reagent (SignaGen) according to the manufacturer’s instructions.

## Northern blot

Total RNA was isolated using Trizol (Life Technologies) and separated in denaturing gels, 20% (w/v) acrylamide with 8 M urea. RNA was transferred to Hybond-N1 membranes (Amersham Pharmacia Biotech), crosslinked and blocked (PerfectHyb™ Plus Hybridization Buffer - Sigma). MicroRNAs were detected using ^32^P-labelled oligonucleotide probes (miR-9-5p: GACTCATACAGCTAGATAACCAAAG, miR-9-3p: GACTTTCGGTTATCTAGCTTTAT). Images were obtained and analyzed using the Amersham Typhoon (GE Healthcare).

## Small RNA NGS library preparation

Total RNA (5 µg) was ligated to the RNA 3’ adaptor using T4 RNA Ligase 2 - truncated (NEB), in the presence of RNase Inhibitor (NEB). RNA 5’ adaptor was ligated using T4 RNA Ligase 1 - high concentration (NEB) and 10 mM ATP. Ligated small RNAs were reverse transcribed using SuperScript® IV Reverse Transcriptase (Thermo-Fisher). The small RNA library cDNA was amplified and indexed using Phusion® High-Fidelity DNA polymerase (NEB). Constructs were purified in a 6% (w/v) native acrylamide gel based on the expected product size and purified by ethanol precipitation. Library quality was assessed using Qubit dsDNA HS Assay Kit (ThermoFisher) and Agilent High-Sensitivity DNA kit (Agilent). Libraries were mixed together and prepared at a final concentration of 12 pM and run on MiSeq Reagent Kit v3 (Illumina) according to the manufacturer’s specifications.

## MicroRNA expression and 5’ isomiR analysis

The primary analysis of miRNA expression and 5’ isomiR analysis were performed using QuagmiR on the NCI Cancer Genomics Cloud [43]. The number of 5’ isomiRs (Nseed) was calculated using an inverse Simpson diversity index [44]. This number measures the evenness of the 5’ isomiRs generated from each individual mature miRNA arm. Of note, Nseed calculation is independent of the annotation of 5’ ends, thus avoiding biases due to multiple and/or misannotations of the 5’ end position. Normal tissue and primary tumour samples from TCGA were used to calculate the number of 5’ isomiRs corresponding to each tumour type. Based on their average expression levels, we selected the most abundant miRNAs (with at least an average of 1 CPM). The high fidelity miRNA group was defined as a set of 30 miRNAs presenting the lowest levels of alternative cleavage across all samples in the dataset (see Supplementary Table 1). To analyze the impact of different RBPs on miRNA processing, we ranked the cohort of BRCA primary tumours according to their FPKM for the selected genes. Two groups of 100 samples with the highest and the lowest expression levels were generated. More details of the R analysis are reported on GitHub (https://github.com/Gu-Lab-RBL-NCI/Drosha-alternative-cleavage).

## Analysis of Microprocessor cleavage fidelity in a library of variants

Data from synthetic libraries of different pri-miRNAs (pri-miR-16-1, pri-miR-30a and pri-miR-125a) were downloaded from GEO accession GSE67937. First, barcoded cleaved fragments data (barcode_cleavage_fragments.txt) were used as 5’ miRNA-offset RNA (5’ moR) to establish the number of cleavage events and the frequency of Drosha cleavage at the canonical and alternative sites. Second, for each barcoded variant of the library (dictionary.txt), the expected minimum-free energy and secondary structure were calculated using the RNAfold from the Vienna package [45]. Subsequent analyses of secondary structures were performed by aggregation of variants of alternative cleavage based on their corresponding dot-bracket notation. More details of the R analysis are reported on GitHub (https://github.com/Gu-Lab-RBL-NCI/Drosha-alternative-cleavage).

## Statistical analysis

The p-values were calculated using the Wilcoxon test as indicated. p-value <0.05 was considered statistically significant. Statistical analysis was performed using RStudio and GraphPad Prism7 statistical software.
